# Unveiling the cardiovascular impact of Zika Virus: Myocardial injury, prothrombotic state, and oxidative stress in immunocompetent mice

**DOI:** 10.1371/journal.pntd.0013292

**Published:** 2025-07-31

**Authors:** Fernanda Marques da Silva, Rafael Aguiar Marschner, Markus Berger, Thais Fumaco Teixeira, Ana Paula Muterle, Letícia Rodrigues, Rafael Teixeira Ribeiro, Paulo Michel Roehe, Diogo Onofre Souza, Carlos Alberto Saraiva Gonçalves, Patrícia Sesterheim

**Affiliations:** 1 Experimental Cardiology Center, Institute of Cardiology/University Foundation of Cardiology of Rio Grande do Sul, Porto Alegre, Rio Grande do Sul, Brazil; 2 Graduate Program in Biological Sciences: Biochemistry, Institute of Basic Health Sciences, Federal University of Rio Grande do Sul, Porto Alegre, Rio Grande do Sul, Brazil; 3 Experimental Research Center, Hospital de Clínicas de Porto Alegre, Porto Alegre, Brazil; 4 Virology Laboratory, Department of Microbiology, Immunology and Parasitology, Institute of Basic Health Sciences, Federal University of Rio Grande do Sul, Porto Alegre, Rio Grande do Sul, Brazil; University of Texas Medical Branch, UNITED STATES OF AMERICA

## Abstract

The Zika virus (ZIKV) has been associated with neurological and cardiovascular complications, including myocarditis, arrhythmias and thrombotic events. This study evaluated the thrombotic and oxidative responses induced by ZIKV in cardiac cells and immunocompetent mice. Cardiac (H9c2) and vascular smooth muscle (A7r5) cell lines were infected with ZIKV and analyzed for viral replication, cytopathic effects and oxidative stress. In vivo, female FVB/N mice were inoculated with ZIKV and cardiac tissue was analyzed for markers of myocardial damage, prothrombotic enzymes and oxidative stress. H9c2 cells demonstrated higher viral replication and cytopathic effects than A7r5 cells. ZIKV-infected cells exhibited increased lactate dehydrogenase release and oxidative stress markers, including elevated protein carbonylation and reactive oxygen species. In vivo, infected mice displayed significant increases in cardiac troponin T levels, indicative of myocardial injury. Analysis of cardiac tissue revealed elevated thrombin and Factor Xa activities and reduced plasmin, indicating a prothrombotic state. Oxidative stress was marked by increased activities of antioxidant enzymes (GPx, SOD) and reduced glutathione levels, alongside heightened protein oxidation. This study demonstrates that ZIKV infection disrupts cardiovascular homeostasis by inducing myocardial injury, prothrombotic state and oxidative stress. These findings underscore the potential of ZIKV to affect the cardiovascular system beyond its established neurotropism, highlighting the need for further investigation into its systemic impacts.

## Author summary

Zika virus (ZIKV) is widely known for its neurological complications, especially in newborns. However, this study reveals that ZIKV can also cause significant effects on the heart. Researchers investigated the impact of ZIKV infection on cardiac cells and healthy immunocompetent mice. They found that the virus is able to replicate in heart cells, leading to cellular damage and oxidative stress, a harmful chemical imbalance in the body. In infected mice, clear signs of heart muscle injury were observed, along with increased levels of clotting-related enzymes, suggesting a higher risk of thrombotic events such as heart attacks or strokes. The study also detected elevated antioxidant responses, indicating the body’s attempt to counteract virus induced stress. These findings demonstrate that ZIKV infection can disrupt cardiovascular function, causing myocardical injury, a prothrombotic state, and oxidative stress. This work expands our understanding of ZIKV’s effects beyond the nervous system and highlights the need for further research into the virus’s systemic impact on human health.

## Introduction

The Zika virus disease outbreak in the Americas during the last decade drew worldwide attention to the neurological impairment caused by infection. The Zika virus (ZIKV) was identified in 1947 in monkeys of the genus Rhesus in the Zika forest of Uganda [1]. The virus was initially endemic to Africa and Asia, but it began to appear with greater incidence in Latin America. Among the countries of South America, Brazil had the highest concentration of infections, and was considered the country with the first significant epidemic of ZIKV, which affected about 1.5 million people [2]. ZIKV is characterized as a neurotropic virus and is associated with congenital complications such as microcephaly, Guillain-Barré syndrome, and meningoencephalitis [3,4]. More recently, clinical neurological manifestations of ZIKV have been observed in adults [5–7], and evidence points to a possible relationship between ZIKV infection and the development of cardiovascular diseases [8–10]. In fact, it has been known since the 1970s that flaviviruses can damage the myocardium, causing the development of myocarditis [11–13]. Case studies indicate that ZIKV can also cause cardiac complications such as arrhythmias, heart failure, fibrillation, myocarditis, hemodynamic impacts, among other clinical manifestations [9,10].

In addition to these complications, the study by Ramaccioti et al., (2019) reports ZIKV-infected patients with elevated D-dimer levels and their potential association with venous thromboembolism [14]. Furthermore, the study by Wu et al., (2017) highlights dysregulation of the coagulation cascade in a ZIKV-infected patient. Additionally, other studies report cases of thrombocytopenia and hemorrhage during infection [15–18], indicating that ZIKV may be associated with severe vascular system alterations. Initially, the intention was to determine if cardiac cells could be infected by a Brazilian strain of ZIKV; to do so, the viral load and viral-induced oxidative stress were measured in those cells. After establishing that the cells were susceptible, and prompted by recent reports suggesting ZIKV could also elicit alterations to coagulation, the next phase was a study in vivo in immunocompetent animals, and during this phase the goal was to assess cardiac tropism of ZIKV, as well as if there are any potential virus-induced changes in the host’s coagulation system, and if infection led to any disruptions in fibrinolysis. In addition, the ZIKV outbreak in Brazil infected thousands of people and led to many microcephaly cases throughout the country. Several studies have uncovered cardiac complications in fetuses infected via vertical transmission [19–21]. In the context of our study, we provide evidence that the Brazilian ZIKV strain not only causes neurological impairments but may also cause and alter cardiovascular complications, even in otherwise healthy subjects.

## Materials and methods

### Study *In Vitro*

#### Ethics statement.

All animal experiments were conducted in accordance with the guidelines of the Brazilian National Council for the Control of Animal Experimentation (CONCEA). The study was approved by the Animal Use Ethics Committee of the Institute of Cardiology of Rio Grande do Sul under protocol number 5705/19.

#### Cells.

H9c2 is a rat’s embryonic cardiac myoblast cell line (ATCC: CRL-1446) obtained from the Cell Bank of Rio de Janeiro, Brazil. A7r5 (ATCC: CRL-1444) are vascular smooth muscle cells (VMSC), obtained from a rat’s thoracic aorta. Vero-E6 (ATCC CRL-1586) are African green monkey kidney cells. Cells were routinely grown in Dulbecco’s modified Eagle’s medium (Gibco, USA), containing 2 mM L-glutamine and 1.5 g/L sodium bicarbonate and supplemented with 10% fetal bovine serum (Gibco) and penicillin/streptomycin (Gibco) at 37°C in a 5% CO2 atmosphere.

#### Viruses.

The ZIKV strain 17SM was isolated in 2016, in São Paulo city, Brazil, from a patient (on day 4 after the first symptoms of Zika). The 17SM ZIKV strain was propagated in Vero-E6. Viral stocks were stored at −80 °C until use. Experiments involving infectious viruses were approved by the Internal Biosafety Commission and conducted in a Level 2 Biosafety Laboratory at the Experimental Cardiology Center, Institute of Cardiology of Rio Grande do Sul (Brazil). Infection of H9c2 and A7r5 cells with ZIKV cells were plated in 24-well plates and infected with ZIKV at different multiplicities of infection (MOI; 1, 0.1, and 0.001) in serum-free medium for 1h at 37°C. Subsequently, fresh DMEM was added, and cells were incubated at different times (24h, 48h, 72h, and 96h) at 37°C. The plates were frozen at -80°C, and the supernatant was used for the analysis of viral load, viral titer, LDH, and oxidative stress.

#### Real-time RT-PCR analysis.

The samples were submitted to total RNA extraction using TRIzol (Ambion), according to the manufacturer’s instructions. The extracted RNA was used for cDNA synthesis with the High-Capacity cDNA Reverse Transcription kit (Applied Biosystems, Thermo Fisher Scientific, 4368813), followed by determination of viral load using quantitative ZIKV PCR (qPCR). The reaction was performed using 2x Platinum SuperMix-UDG quantitative PCR mix (Invitrogen - Life Technologies, 11730017), with 200 nM of each forward (ZIKV 1086) and reverse primer (ZIKV 1162c) [22]. Amplification was carried out on the StepOne Real-Time PCR system (Applied Biosystems, Thermo Fisher Scientific_ under the following conditions: incubation with uracil DNA glycosylase at 50°C for 2 minutes, initial denaturation and Platinum Taq activation at 95°C for 2 minutes, followed by 40 amplification cycles (15 second at 95°C and 30 seconds at 60°C). The viral RNA quantification was estimated relative to a standard curve for ZIKV (ranging from 10^8–10 copies), and data analysis was performed using StepOne software v2.2.2.

#### Virus particle labeling (PKH26) and cell infections.

The PKH26 dye was diluted in 2 mL of Diluent C (kit MINI26; Sigma‒Aldrich Co., USA), immediately before staining. One volume of ZIKV suspension plus two volumes of PKH26 were mixed by pipetting in an Eppendorf [23]. After 1 minute, virus labeling was stopped by adding three volumes of DMEM with serum.To infect MOI 1 of ZIKV with a 1x107 titer was used, and cells were plated in 24-well plates, 3x10^5^ cells per well. The cells were infected and incubated for 1h at 37°C in an atmosphere of 5% CO2; after the adsorption period, the supernatant was removed, and 500 µl of DMEM with serum was added. The sample was fixed, after 24h, by removing the DMEM and adding 200µl of paraformaldehyde 4%. Samples were kept at 4°C for 24h. After the removal of paraformaldehyde, Hoechst dye was added, and cells were incubated for 2 minutes on a shaker. Excess dye was removed by washing with PBS. Samples were covered with glass coverslips and analyzed using a confocal laser scanning Olympus FluoView 1000 microscope.

#### Lactate dehydrogenase activity.

The LDH activities in the supernatants of cells (MOI 1) that had been infected for different periods (24, 48, 72, and 96 hours) were determined using a kit from Sigma Aldrich (MAK066) in which LDH reduces NAD to NADH, which is specifically detected by a microplate colorimetric assay. The initial absorbance was measured at 450 nm (A450).

#### NBT reduction.

Cells were incubated with 0.1% nitroblue tetrazolium (NBT) (1mg/ml) at 37°C in an atmosphere of 5% CO2 for 1 hour. The NBT was removed, and the cells were washed with 1x PBS and then with 70% methanol, followed by quenching with 100% chilled methanol. Potassium hydroxide (KOH; 2M) and DMSO were used to dissolve the crystals. Absorbance was measured at 620 nm using a Synergy H1 microplate reader.

#### Protein oxidation.

The protein carbonyl concentration was determined according to the method of Levine et al. (1994) [24]. Samples were briefly homogenized in 1 mL of 1 M Tris-HCl buffer containing 50 mM EDTA, 100 mM phenylmethane sulfonyl fluoride (PMSF), and 100 mM Benzamidine. The homogenate was centrifuged at 11 000 g for 15 min at 4°C. Supernatants (500 µl) were mixed with 10% (w/v) streptomycin sulfate for 15 min to remove nucleic acids. After further centrifugation, the supernatants were mixed with 500 ml of 20% TCA for an additional 15 minutes. The supernatants were discarded, and the precipitates were dissolved in 500 µl of 10mM 2,4 dinitrophenylhydrazine (DNPH) in 2M HCl. The samples were incubated for 1h at room temperature, the proteins were precipitated with 20% trichloroacetic acid (TCA), and, afterward, the sediments were washed three times with 1 ml of ethanol. The pellets were dissolved in 6 M urea, and absorbance was measured at 370 nm using a spectrophotometer. The carbonyl concentration was determined using the molar absorption coefficient for aliphatics and expressed in nmol carbonyl/mg for protein.

#### In Vivo Study.

***Animals***. The use of female mice in this study was chosen due to the biological and epidemiological relevance observed during the 2016 ZIKV epidemic in Brazil, when a significant number of women were affected. Women constitute a particularly vulnerable risk group, as ZIKV infection was associated with severe fetal complications, including microcephaly and other congenital abnormalities [2–4]. Consequently, this experimental model offers a more representative framework for investigating the mechanisms of pathogenicity and the pathophysiology of the infection. Therefore, female mice were utilized in this study. Ten-week-old FVB/N female mice were obtained from the Cardiology Experimental Center (Institute of Cardiology of Rio Grande do Sul, Brazil). Mice were housed at the Animal Research Facility under a 12h light, 12h dark cycle at a constant temperature of 22 ± 2 °C with free access to commercial chow and water.

#### Mice intraperitoneal ZIKV infection and sample collection.

Eighty-day-old female mice (n = 8) were inoculated with 105 PFU of ZIKV-BR intraperitoneally in a final volume of 100 µl. The animals of the control group (n = 8) were inoculated intraperitoneally with 100 µl of DMEM without serum. After 96 hours of infection (hip), spleen collection was performed and the animals were later euthanized for collection of the cardiac tissue, which was stored at -80°C for later analyses. The 96-hour post-infection time point was determined in a pilot study as the optimal period, as viral infections are well-established, the immune response is active, and tissue damage is detectable.

#### Real-time RT-PCR analysis and virus isolation.

Real-time RT-PCR assays were performed in triplicate, following the same protocol used in the in vitro study [22].

#### Quantification of cardiac troponinT (TnT).

The submandibular mouse vein was used as a blood source (96 hours post-infection) for quantification of cardiac troponin T (TnT) on an Elecsys 2010 immunoassay analyzer (Roche Diagnostics, Mannheim, Germany).

#### Analysis of the activity of prothrombotic and fibrinolytic enzymes in cardiac tissue.

Hearts were dissected from euthanized animals, macerated into small pieces (approximately 1mm), and washed in Ringer’s solution. Subsequently, tissue was homogenized in RIPA buffer in the absence of protease inhibitors, using an Ultra Turrax type homogenizer. Later, the samples were centrifuged at 12 000 rpm/4°C, and the supernatant was collected. The dosage of total protein was made using the bicinchoninic acid (BCA) method. For acceleration of color development, samples were incubated at room temperature for two hours, at 37°C for 30 minutes, and at 600°C for 30 minutes. After incubation, samples were cooled to room temperature, and 580 nm absorbance measurements were read immediately. A standard curve was formed with bovine albumin (BSA), which was the standard solution included in the kit. The same amount of protein was used for all samples (30 µg), and specific chromogenic or fluorogenic substrates were employed for each enzyme (thrombin, plasmin, vasopressin, kallikrein, activated factor X (FXa) and aminopeptidases) [25].

For the chromogenic FIX activity assay, the first stage consisted of mixing the test plasma with FXIa, FVIII, thrombin (FIIa), prothrombin (FII), FIX, phospholipids, and calcium, which led to FIX-dependent activation of FX (FXa). During the second stage, an off-target enzyme inhibitor (not FXa) and an FXa-specific chromogenic substrate were added, and the FXa activity levels were quantified by monitoring FXa cleavage of the chromogenic peptide substrate [25]. The kinetics of each reaction were accompanied by measuring the release of p-nitroaniline (at 405 nm) or β-naphthylamine (360 nm/ 460 nm) using a 96-well microplate spectrophotometer. The results were expressed in terms of the speed at which enzyme activity (m0D/ min) was normalized according to the amount of protein found in each well.

#### Analysis of protein carbonylation.

The difference between samples treated with 2,4-dinitrophenylhydrazine and treated with HCI (white) was used to calculate the carbonyl content, determined at 370 nm [26]. The data obtained was calculated using the absorption coefficient millimolar of hydrazine (e370 nm = 21.000000.M-1 cm-1), and the results were expressed in nmol carbonyl/mg of protein.

#### Reduced glutathione (GSH) levels.

GSH levels were measured according to the method described by Browne & Armstrong (1998) [27]. Absorbance was measured at 420 nm in a microplate reader using reduced glutathione as an external standard (0.001–1 mM). Results were expressed as nmol/mg protein.

#### Glutathione peroxidase activity.

The glutathione peroxidase (GPx) assay was performed according to Wendel (1981) [28], using tert-butylhydroperoxide as a substrate. The activity-specific gravity was calculated as U per milligram of protein.

#### Superoxide dismutase activity.

Heart samples were homogenized in PBS at pH 6,5 at a ratio of 1:10 (mg/ml) in a Polytron homogenizer (9 700 g, 20 min, 4°C). Superoxide dismutase (SOD) activity was measured as the ability of the enzyme to inhibit pyrogallol self-oxidation. The enzyme concentration that inhibited the reaction by 50% (IC50) was defined as one unit of SOD, expressed as SOD activity unit per mg of total protein (units SOD/per mg protein) [29].

#### Statistical analyses.

The data was presented as means ± SD, and significant differences were analyzed using one-way ANOVA, followed by an unpaired t-test with a Bonferroni correction for multiple comparisons. P-values < 0.05 were considered to be significant. Statistical analyses were performed using GraphPad Prism v.6 (GraphPad Software Inc., San Diego, CA, USA).

## Results

**Fig 1:**
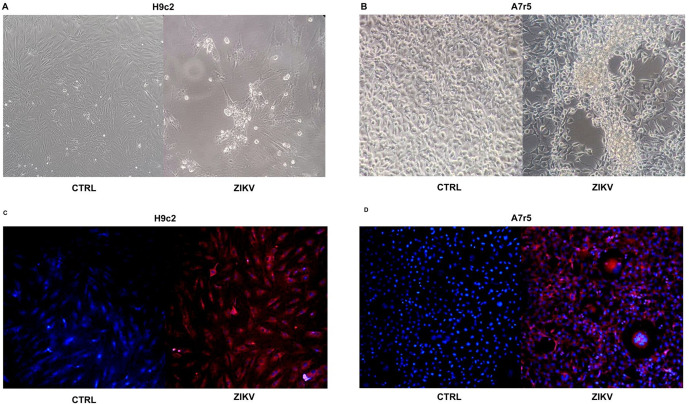
Cytopathic effect and internalization of th ZIKV in cell lines. In panels A) and B) (H9c2 and A7r5 cell lines phase contrast, magnified 40x) before (upper panels) and 72 h after of ZIKV exposure, arrowheads indicate morphological alterations. In C) and D), representative confocal images of PKH26-positive ZIKV viral particles in H9c2 and A7r5 cell lines. PKH26-positive cells (in red) and DAPI-stained nuclei (in blue) 1 h after ZIKV infection (magnified 20x). See details in Material and Methods.

**Fig 2:**
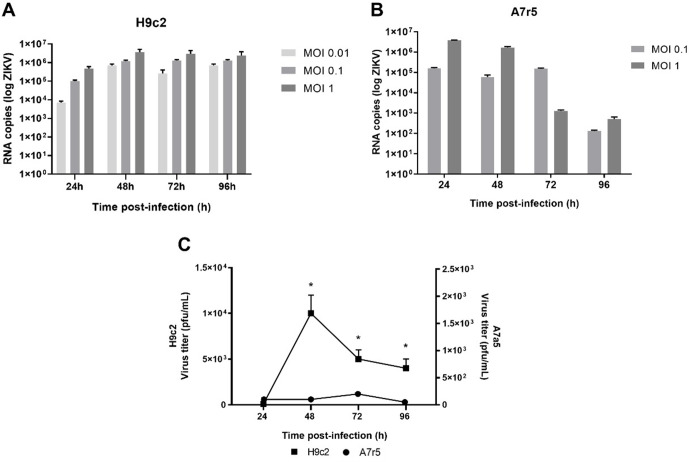
Susceptibility and permissiveness to ZIKV-BR (MOI1) infection in cardiac cell lines. In A) and **B)**, ZIKV viral load quantification in H9c2 and A7r5, respectively (N = 3 per group). In **C)**, quantification of infectious ZIKV particles by plaque assay in infected cells with MOI 1 (N = 3 per group). Statistical analysis was performed by Student t test in relation to its respective control, assuming *p < 0.05 and **p < 0.01.

**Fig 3:**
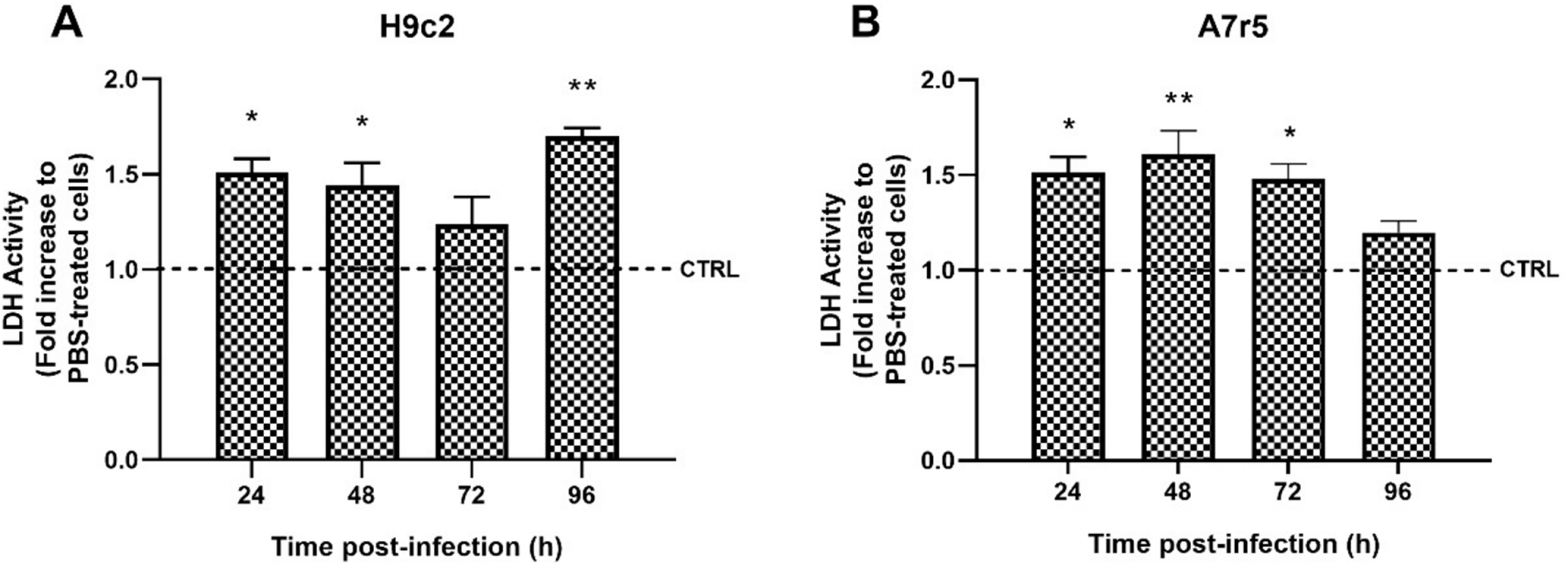
Extracellular lactate dehydrogenase activity in ZIKV-infected cell lines. H9c2 A) and A7r5 B) cell lines, the dashed line indicates the control value and the data represent the mean ± standard deviation of the experimental samples (N = 3 per group). Statistical analysis was performed by Student t test in relation to its respective control, assuming *p < 0.05 and **p < 0.01.

The H9c2 and A7r5 cell lines presented cytopathic characteristics of viral infection (Fig 1A and 1B); it was possible to observe cell rearrangement, with minimal morphological alteration, showing vacuolization and syncytial organization (cytopathic effect, CPE). The adsorption and internalization of the virus by the infected cells was confirmed using the fluorescent marker PKH26 (Fig 1C and 1D). Molecular analysis indicates that cell cultures were susceptible to ZIKV, leading to productive infection and the release of infectious viral progeny (Fig 2A and 2B). During the analysis of the formation of viral infective particles, the H9c2 strain was found to produce a significantly higher amount of virus than the A7r5 strain, in which, although viral genomic RNA was detected, it was not possible to verify a significant increase in viral titer over time (Fig 2C and 2D). Thus, these results demonstrate that H9c2 cells are susceptible to ZIKV infection, which is capable of efficiently multiplying in these cells, reaching satisfactory titers. Based on the extracellular LDH assay, partial losses of cellular integrity were observed in H9c2 and A7r5 (Fig 3A and 3B, respectively) 24h post-infection, and this was maintained up to 96 h.

**Fig 4:**
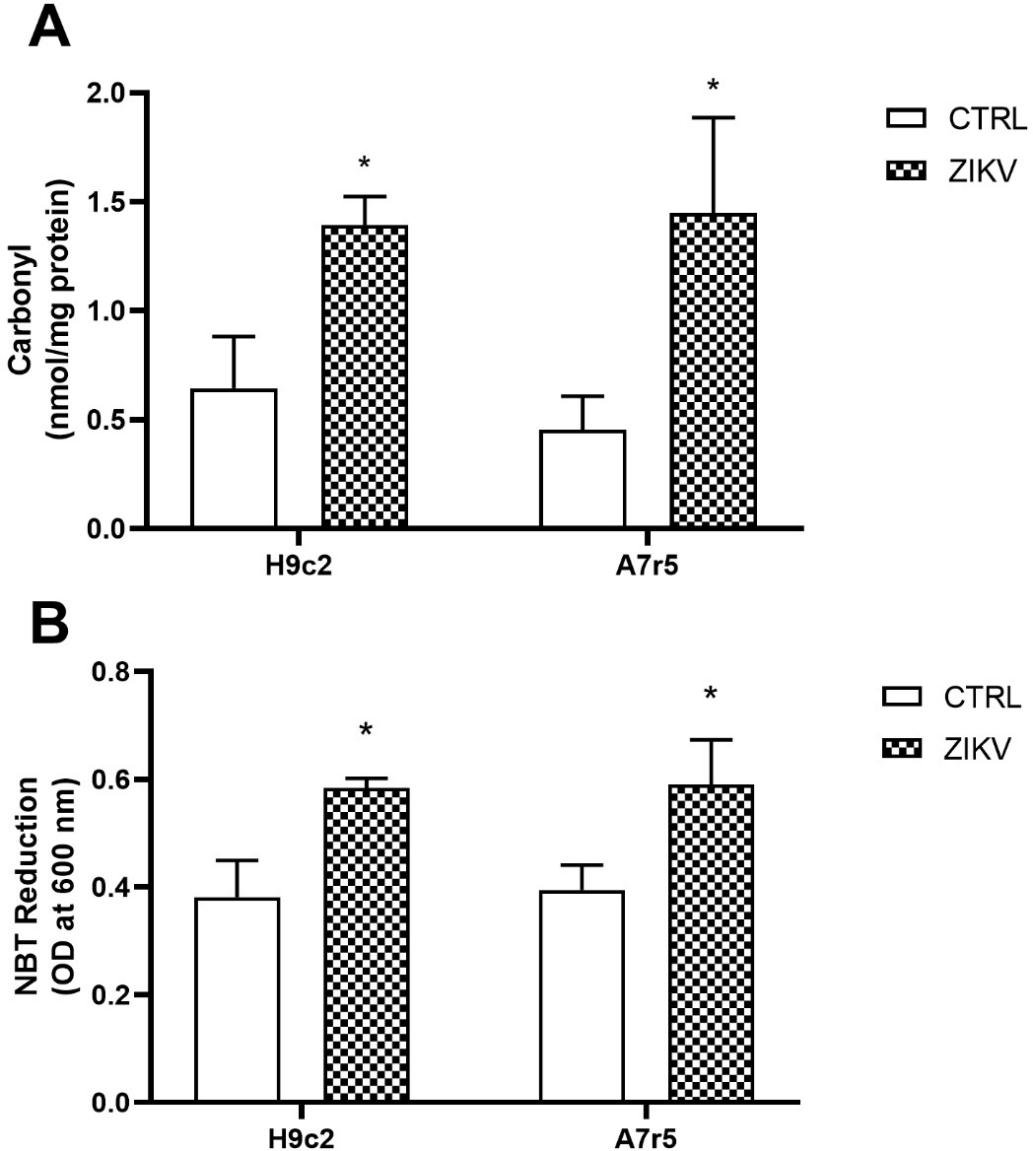
Oxidative stress in H9c2 and A7r5 ZIKV-infected cell lines. In A), protein carbonylation. Values are expressed as mean ± standard deviation and presented as carbonyl nanomoles/mg protein (N = 3 per group). In B), NBT reduction assay as an index of superoxide anion production (N = 3 per group). Statistical analysis was performed by Student t test in relation to its respective control, assuming *p < 0.05.

The production of reactive oxygen species (ROS) is part of the inflammatory process. Thus, we analyzed cellular oxidative stress by evaluating levels of protein oxidation (protein carbonylation) in infected cells. ZIKV infection resulted in a significant increase in carbonyl content in both cell lines (Fig 4A) and increased superoxide production, as shown by the NBT photoreduction assay (Fig 4B).

**Fig 5:**
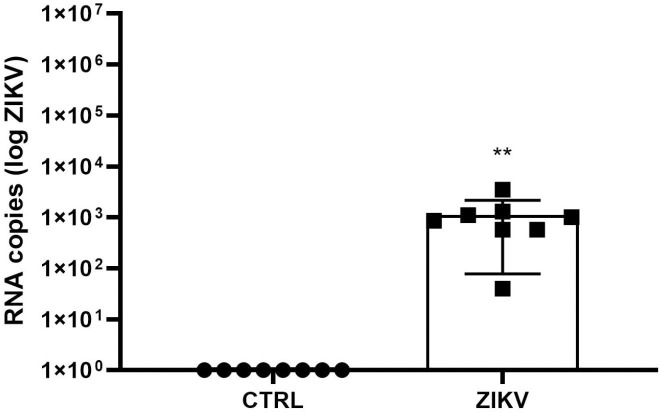
Splenic and cardiac ZIKV detection in infected mice. Viral genome detection in the splenic tissue of FVB/N mice at 24 h after inoculation with 105 PFU of ZIKV. The data presented had a mean ± standard deviation of 1.12 x 103 ± 1.04 x 103 log10 copies/mL (N = 8 per group), assuming **p < 0.01.

**Fig 6:**
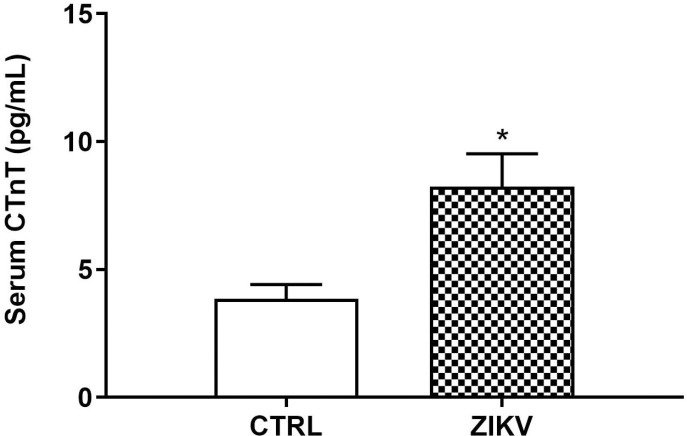
Serum troponin T in ZIKV-infected mice. Troponin T (Tn T) levels in the serum of immunocompetent control and ZIKV-infected animals. See details in Material and Methods. Data are represented as mean ± standard error. Statistical analysis was by Student t test where the significant values observed were *p < 0.05 (N = 8 per group).

**Fig 7:**
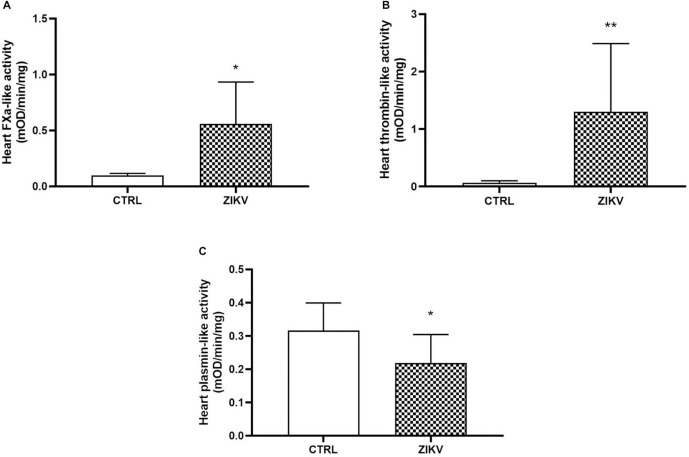
Cardiac enzymes of coagulation and fibrinolysis in ZIKV-infected mice. In **A)**, cardiac levels of factor Xa; in **B)**, cardiac levels of thrombin; and, in **C)**, levels of plasmin. Quantitative assays were based on their activity on specific chromogenic substrates. See details in Material and Methods. Data are represented as mean ± standard error. Statistical analysis was by Student t test where the significant values observed were *p < 0.05 (N = 8 per group) and **p < 0.01.

In the in vivo study, female FVB/N mice were used to mimic the patients most affected by the ZIKV epidemic in Brazil in 2015. Additionally, these animals are immunocompetent, allowing us to investigate the potential damage that ZIKV may cause in healthy individuals. We collected and analyzed the spleen and heart from FVB/N mice, 24 hours after inoculation with 105 PFU of ZIKV. The RT-PCR analysis revealed that only the spleen of infected females tested positive and had a viral load of 1.12 x 103 ± 1.04 x 103 (mean ± deviation) Log copies/mL, respectively (Fig 5). Serum levels of cardiac troponin T (TnT) were significantly higher at 96 hours post-infection in infected animals, possibly indicating the occurrence of myocardial injury after ZIKV infection (Fig 6; p < 0,05) [30]. When analyzing procoagulant and fibrinolytic enzymes, Xa factor and, consequently, thrombin were found to be significantly elevated in the group of infected females (Fig 7A and 7B; p = 0.0226 and p = 0.0065, respectively). On the other hand, the presence of the ZIKV virus in females led to a decrease in the plasmin enzyme responsible for fibrin degradation (Fig 7C; p = 0.0351), indicating an imbalance between procoagulant and profibrinolytic factors, leading to a prothrombotic state [14].

**Fig 8:**
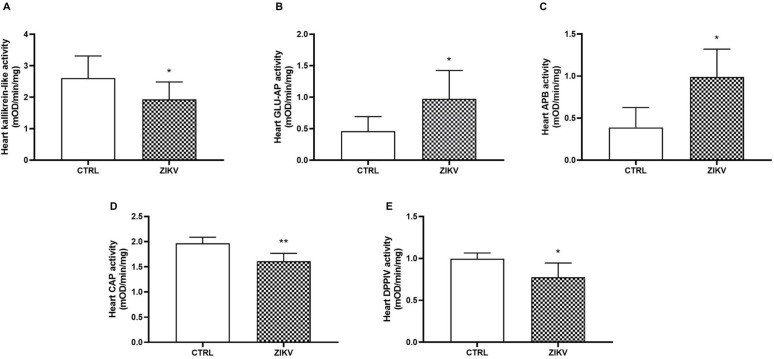
Kallikrein and aminopeptidases activities in cardiac tissue of ZIKV-infected mice. In A), Kallikrein; In B), A aminopeptidase (GLU-AP); In C), B aminopeptidase (BAP); In D), cystine aminopeptidase (CAP); and in E), dipeptidyl peptidase IV (DPPIV). See details in Material and Methods. Data are represented as mean ± standard deviation. Statistical analysis was performed using the Student t test, assuming *p < 0.05 as significant (N = 8 per group).

Moreover, kallikrein levels in cardiac tissue were reduced in the ZIKV group, when compared to the control group (Fig 8A; p = 0.0338). The activity of aminopeptidases, responsible for converting angiotensin II into III (GLU-AP, aminopeptidase A, glutamil-aminopeptidase) and angiotensin III into IV (APB, aminopeptidase B), was increased in the presence of the virus (Fig 8B and 8C; p = 0.0320 and p = 0.0111, respectively). However, cystine aminopeptidase (CAP), the degrading vasopressin enzyme (Fig 8D; p = 0.0037), exhibited a decrease in activity in the cardiac tissue of infected animals. Moreover, the DPPIV activity (Fig 8E; p > 0.0151) in the cardiac tissue of infected animals was significantly lower compared to the controls, indicating potential alterations in cardiac function and blood pressure regulation. These findings suggest a hypertensive state in these animals after ZIKV infection.

**Fig 9:**
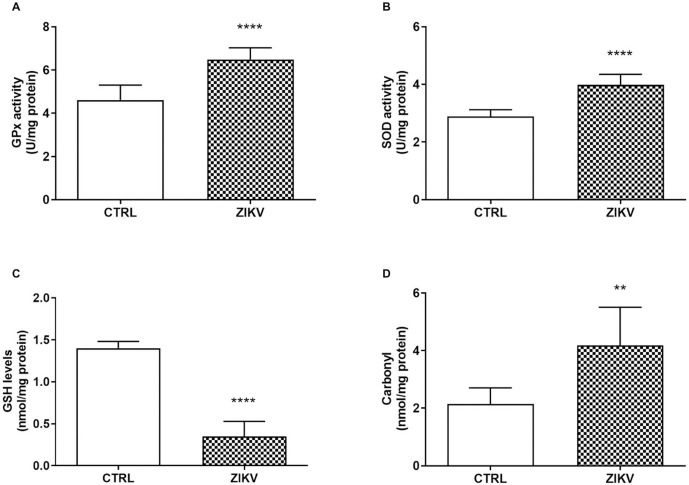
Oxidative stress in cardiac tissue of ZIKV-infected mice. In A) (GPx) activity; In B), superoxide dismutase (SOD activity); In C), reduced glutathione (GSH) levels; and In D), protein carbonylation levels. See details in Material and Methods. Data are expressed as mean ± standard deviation. Statistical analysis was by Student t test where the significant values observed were *p < 0.05 (N = 8 per group), **p < 0.01, ****p < 0.0001.

As observed in vitro, the animals also presented significant levels of oxidative stress after ZIKV infection. There was an increase in GPx and SOD enzyme activities in the cardiac tissue of infected animals (Fig 9A and 9B, respectively), as well as a decrease in glutathione levels (Fig 9C). ZIKV infection also increased protein carbonylation in the cardiac tissue (Fig 9D) (p < 0,0001). The results demonstrate the important role of ZIKV in disrupting homeostasis in cardiac cells in mice, leading to a state of oxidative stress and the recruitment of antioxidant enzymes to the site of infection.

## Discussion

The current study illustrated that ZIKV, in addition to infecting cardiac cells and causing damage, is also capable of infecting immunocompetent animals, resulting in cardiac tissue injury dysfunction in the coagulation cascade, vasodilation, and a significant increase in reactive oxygen species, indicating substantial injury and failure in antioxidant regulation. Based on available evidence, no prior studies have investigated the effect of ZIKV infection on blood coagulation homeostasis in immunocompetent animals.

Different cell lineages are known to be susceptible to ZIKV infection [31]; however, the susceptibility and permissiveness of cardiac cells have only recently become the subject of studies [32]. From this perspective, the susceptibilities of a mouse embryo myoblastic cell line (H9c2) and a murine aortic smooth muscle cell line (A7r5) to ZIKV were investigated. A study by Ambrocio et al. (2015) demonstrated that H9c2 cells were susceptible to the dengue virus [33]. Likewise, in our study, we detected the viral genome and an increase in the viral titer for 96 hours post-infection. Additionally, Rossi et al. (2020) investigated ZIKV infection in human cardiac mesenchymal stromal cells that were extracted from fetuses at embryonic age [32]. They observed that the cells were susceptible to the virus, but there was a significant viral reduction at 96 hours post-infection, whereas in our study, there was an increase in the viral titer in H9c2 cells even during the first 48 hours.

In this context, when we evaluated the susceptibility and permissiveness of A7r5 cells, we observed that the viral genome was detected at high loads. During the periods evaluated, the cells released genome copies into the extracellular environment; however, the process of maturation of viral particles did not occur as expected. A similar phenomenon was reported during dengue infection (Arias-Arias et al., 2018), possibly causing the low viral titer of ZIKV observed in A7r5 lineage cells, and indicating a problem with viral morphogenesis in these cells [34]. The various stages of the viral replication cycle interfere with the homeostasis of cellular metabolism, triggering an imbalance in the redox state in host cells. Some studies have shown changes in the redox state associated with the inflammatory process related to ZIKV infection in neurons and astrocytes [35,36]. The results from this study clearly show the induced pro-oxidative redox imbalance by ZIKV infection in cardiomyoblasts and murine aortic smooth muscle cells. In fact, most of the protein structure of members of the Flaviviridae family focuses on inducing oxidative stress, which would explain the possible tissue damage after infection, or even the induction of autophagy in infected cells [37,38].

Given the results obtained in the in vitro study, there was a need to investigate the potential damage caused by ZIKV infection in an in vivo model. Thus, the cardiac damage caused by ZIKV was confirmed in our infection model, where FVB/N mice infected with ZIKV showed elevated serum levels of the myocardial injury marker (TnT). Other studies have used knockout mice for the IFNa/b receptor as an animal model for ZIKV infection and found elevated levels of TnT, as well as increased cardiac muscle enzymes, suggesting acute myocardial injury [30,39]. Here, the emphasis is on the fact that the model of healthy immunocompetent animals also showed significant results for cardiac damage, as well as dysfunctions in the coagulation cascade and oxidative stress.

Factor Xa is a serine protease involved in converting fibrinogen to insoluble fibrin, forming a clot, as well as playing a key role in the enzymatic sequence that culminates in the production of a thrombus. We verified an activation of procoagulant enzymes in infected hearts and a negative regulation of fibrinolysis, indicating a prothrombotic state. In addition, serine proteases (e.g., factor Xa and thrombin) are capable of activating protease-activated receptors in cardiac cells, triggering inflammation and amplifying coagulation signaling. Downregulation of kallikrein during ZIKV infection also indicates a reduction in kinin activity, since this is the main enzyme capable of releasing bradykinin. These data corroborate a study that observed an increase in D-dimer and dysfunction of the coagulation cascade after ZIKV infection [14], as well as another investigation that found increased blood pressure and prothrombin in infected individuals [40]. As such, the mechanism behind this imbalance in coagulation and vasodilation processes after ZIKV infection is still unclear, but other flaviviruses, such as dengue and yellow fever virus, are also known to modulate coagulation factors, resulting in atypical clots, bleeding and thrombosis [41–45], which may cause vascular accidents and amyloidogenesis [46].

The results obtained in this study confirm the significant impact of ZIKV infection on oxidative stress in the cardiac tissue of mice, reinforcing the hypothesis that ZIKV may cause damage to organs beyond the central nervous system, traditionally described as the virus’s primary target. The increase in the activities of the antioxidant enzymes GPx and SOD suggests a compensatory response by the body’s antioxidant system to the elevated levels of reactive oxygen species (ROS) induced by the infection [47]. GPx and SOD, which convert superoxide radicals into hydrogen peroxide, were significantly activated in the infected cardiac tissue, indicating a cellular response to oxidative insult [48]. However, the reduction in GSH levels may indicate the depletion of antioxidant resources in response to the excessive accumulation of ROS. GSH is one of the major intracellular antioxidants, and its depletion suggests that the endogenous antioxidant system was insufficient to mitigate the oxidative stress induced by the infection. GSH depletion is frequently associated with cellular dysfunction, which can ultimately lead to tissue damage and impaired cardiac function [49].

Previous studies have suggested an association between oxidative stress and various cardiac pathologies, such as cardiac hypertrophy, fibrosis, and heart failure. Although it remains unclear whether ZIKV infection can directly cause heart disease in humans, our results suggest that the virus is capable of affecting cardiac tissue in experimental models, inducing a pro-oxidative environment that contributes to cardiac dysfunction. This study underscores the need for further investigation into the mechanisms through which ZIKV induces oxidative damage in peripheral organs and the long-term impact of this infection on cardiac tissues.

## Conclusions

This study indicates that the Brazilian ZIKV strain possesses cardiac tropism, infecting myoblasts (H9c2 cells) and vascular smooth muscle cells (A7r5), although each cell type exhibits distinct viral replication characteristics. In this immunocompetent in vivo model of ZIKV infection, direct cardiac injury occurred, as demonstrated by elevated serum troponin T values, disruption of antioxidant mechanisms, including depletion of glutathione and compensatory activation of GPx and SOD enzymes, and the identification of a prothrombotic state. The prothrombotic state was exhibited via: (i) activation of procoagulant enzymes, such as factor Xa, (ii) downregulation of fibrinolysis, and (iii) vasoactive disturbances associated with protease activated receptor modulation and decreased kallikrein-kinin system functionality.

The current findings greatly expand the known pathogenic possibilities of ZIKV (historically considered to cause neurological sequelae) by demonstrating ZIKV may induce viral cardiomyopathies and thromboembolic states in an immunocompetent host. The observed cardiovascular changes share similarities with other flaviviruses (e.g., dengue or yellow fever viruses) and may indicate that similar mechanisms of vascular injury exist within this family of viruses. However, more work is needed to evaluate the specific molecular pathways employed by the ZIKV specifically.

The data show the importance of cardiovascular monitoring of ZIKV-infected patients, especially where pre-existing comorbidities are present, from a translational perspective. On the other hand, our data also serve as a rationale for potential clinical studies assessing the use of adjuvant therapies (such as anticoagulants and antioxidants) for severe patients. This work provides an experimental foundation to continue studying the long-term cardiac sequelae of ZIKV infection and the development of definitive therapeutic approaches.

### Study limitations

First, even though we employed cell lines from rats (H9c2/A7r5) for in vitro studies, the infection model was ultimately developed using mice (FVB/N) because the approach failed in rats (The rats were persistently negative for ZIKV by PCR). This difference in taxonomic relevance between in vitro and in vivo systems limits direct extrapolation, but both in vitro and in vivo systems utilize mechanisms that are conserved in cardiovascular pathways. Second, even though we characterized the acute cardiac injury (96h after inoculation with viral particles), we did not address the consequences of ZIKV over time. Third, mechanistic connections need to be examined with genetic approaches using both oxidative stress and coagulopathic events in relation to the specific viral proteins observed. Lastly, these significant in vivo results were produced from FVB/N mice, whereas further studies could include organisms with humanized mice or alternative strains to increase clinical relevance. Despite the aforementioned limitations, our experimental system set forth a validated starting point for preliminary mechanistic studies. Also, murine animal models have a distinct advantage to immunological characterization that would be challenging to ethical parameters in humans.

## Supporting information

Supporting information (ZIP)
